# Supplementary material for: Spatial heterogeneity and infection patterns on epidemic transmission disclosed by a combined contact-dependent dynamics and compartmental model
Source: PLoS One. 2023 Jun 13;18(6):e0286558. doi: 10.1371/journal.pone.0286558 (PMC10263307; doi:10.1371/journal.pone.0286558)
Supplement: S1 File — (DOCX) [file pone.0286558.s001.docx]

**Spatial heterogeneity and infection patterns on epidemic transmission disclosed by a combined contact-dependent dynamics and compartmental model**

Youyuan Zhu^a,b^, Ruizhe Shen^a,b^, Hao Dong^a,c,d,^*, & Wei Wang^b,c,^*

^a^ Kuang Yaming Honors School, Nanjing University, Nanjing 210023, China;

^b^ Collaborative Innovation Center of Advanced Microstructures, National Laboratory of Solid State Microstructure, & Department of Physics, Nanjing University, Nanjing 210093, China;

^c^ Institute for Brain Sciences, Nanjing University, Nanjing 210023, China.

^d^ State Key Laboratory of Analytical Chemistry for Life Science, Nanjing University, Nanjing 210023, China.

* Corresponding author

E-mail: [donghao@nju.edu.cn](mailto:donghao@nju.edu.cn) (HD)

E-mail: [wangwei@nju.edu.cn](mailto:wangwei@nju.edu.cn) (WW)

**Computational Details**

**1 Contact-dependent dynamics with no contact (CDD-NC) model**

In the CDD-NC model (**S1 Fig a**), the velocities and directions of the individuals follow the same distribution in CDD, and the motion does not change when two particles are in contact. As shown in **S1 Fig b**, we also estimated the exposure time ***ω*** (the time that two particles stay in contact) in both the CDD and CDD-NC models. We found that there was nearly no difference between the two models at high velocity with respect to the distribution of ***ω***. At low velocity, agents prefer short-term contacts at low velocity in the CDD model.

**
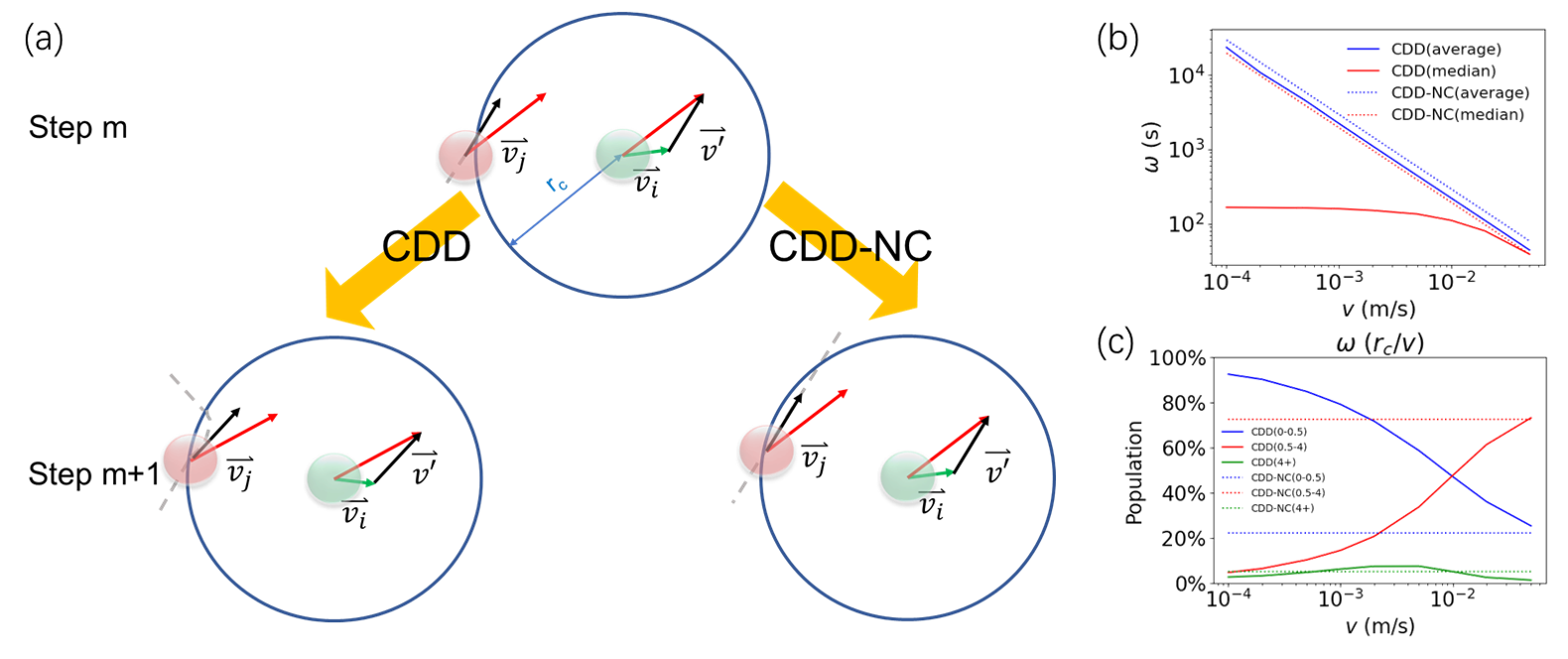
**

**S1 Fig. Comparison of the CDD model with the CDD-NC model.** (a) Schematic diagram of the CDD model and the CDD-NC model. ***i*** is the central agent, and the blue circle represents its contact range of radius ***r_c_***. Individuals ***i*** and ***j*** contact when the distance between the two is less than ***r_c_***. The red, green, and black solid vectors represent the velocities of ***i*** and ***j*** and the relative velocity ***j*** relative to ***i***, respectively. The gray dashed line represents the trajectory of ***j*** relative to ***i***. (b) The mean (blue) and median (red) of the contact time ***ω*** influenced by the velocity ***v*** of agents in the CDD model (solid line) and CDD-NC model (dashed line). (c) The percentages of ***ω*** in different time ranges for the velocity ***v*** of agents in the CDD model (solid line) and CDD-NC model (dashed line). The blue line represents ***ω*** < 0.5****r_c_*** /***v***, the red line represents 0.5****r_c_*** /***v*** ≤ ***ω*** < 4****r_c_*** /***v***, and the green line represents ***ω*** ≥ 4****r_c_***/***v***.

**2 The exposure time *ω* and mean free time *τ***

**2.1 The exposure time *ω***

**CDD model:** We record the start and the end time (***ω_s_*** and ***ω_e_***, which are in the unit of seconds) of each contact in all trajectories in which agents move with a mean velocity ***v***. The exposure time ***ω*** (the time that two agents contact) is calculated by ***ω***=***ω_e_***-***ω_s_***+1. Then, we will carry out statistical analysis on ***ω***.

**CDD-NC model:** We set a Cartesian coordinate system. The horizontal and vertical axes are referred to as the ***X***-axis and ***Y***-axis, respectively. A central agent ***a*** is set on the origin ***O***, and agent ***b*** is the neighbor of ***a***. The velocities of agents ***a*** and ***b*** (***v_a_*** and ***v_b_***) follow the normal distribution with a mean of ***v*** and a standard deviation of $\frac{1}{3}\boldsymbol{v}$. The directions ***θ_a_*** and ***θ_b_*** are randomly generated from a uniform distribution. The distributions are the same as those in the CDD model.

*
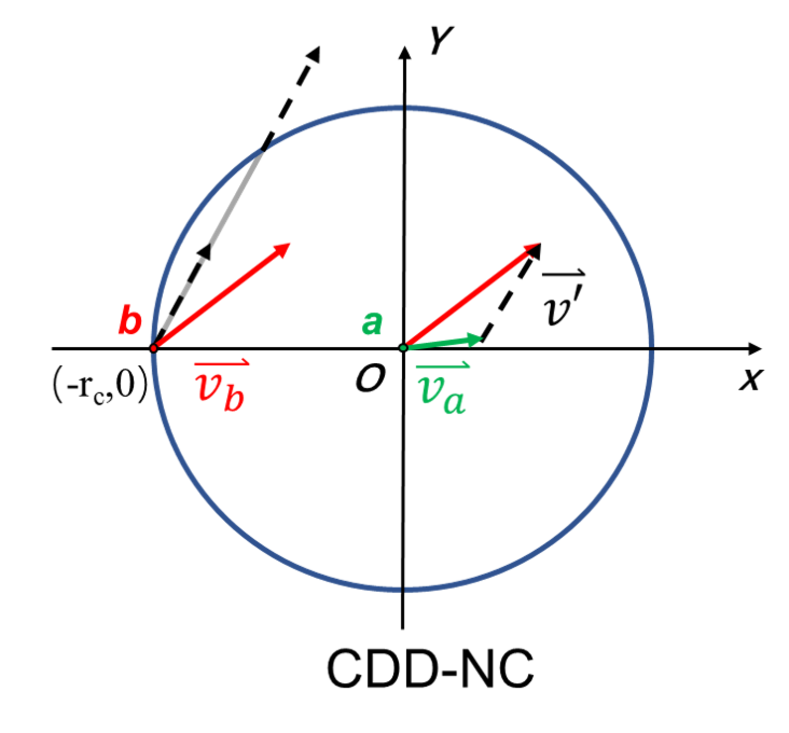
*

**S2 Fig. Schematic diagram of the CDD-NC model for the calculation of the exposure time *ω.*** The blue circle represents the contact range of radius ***r_c_***. The red and green solid vectors and black dashed vector represent the velocities of ***a*** and ***b*** and the relative velocity ***b*** relative to ***a***, respectively. The gray solid line represents the trajectory of ***b*** relative to ***a***.

We assume that agent ***b*** approaches agent ***a*** from (-***r_c_***,0). The relative velocity of ***b*** with respect to ***a*** is ***v´***. The ***x*** and ***y*** components of ***v´*** are ***v_x_´*** and ***v_y_´*,** respectively. The exposure time ***ω*** is the time that agent ***b*** passes through the contact range of agent ***a***. We run this simulation over 200000 times and calculate ***ω*** by equation 1 when ***v_x_´***>0. Then, we will carry out statistical analysis on ***ω***.

$\omega=\frac{2r_{c}cos<\vec{v}^{'},\vec{e_{x}}>}{v^{'}}=\frac{2r_{c}v_{x}^{'}}{{\vec{\vec{v}^{'}}}^{2}}$ (1)

where $\vec{e_{x}}$ represents the unit vector of the ***X***-axis and $<\vec{v}^{'},\vec{e_{x}}>$ represents the angle between $\vec{v}^{'}$and $\vec{e_{x}}$.

**2.2 The mean free time *τ***

**CDD model:** We record the number of contacts ***T_i_*** in the ***i***th trajectory in which agents move with a mean velocity ***v***. We calculate the mean free time ***τ*** by equation 2 over 5 trajectories.

$\tau=\frac{N*40day*5}{\sum_{i} T_{i}}$ (2)

**CDD-NC model:** As shown in **S2 Fig**, we run the simulation over 200000 times and calculate the average relative velocity ***b*** relative to ***a*** $\bar{v’}$=1.35***v***. We estimate the mean free time ***τ*** by equation 3.

$\tau=\frac{1}{2r_{c}\bar{v'}\rho}$ (3)

**3 The basic reproduction number *R_0_***

**3.1** By derivations in the main text, we calculated the basic reproduction number ***R_0_*** in the CDD-SEIR model by

$R_{0}=\frac{N}{p_{IR}\Delta t}<\frac{{\Delta N}_{S}}{N_{S}N_{I}}>$ (4)

where ***N***, ***N_S_*** and ***N_I_*** represent the total, susceptible and infectious populations of agents, respectively, and ***ΔN_S_*** represents the variation of ***N_S_*** in the integration time step ***Δt***.

**3.2** We calculated the basic reproduction number ***R_0_*** by equation 5 over all 25 simulations.

$R_{0}=\frac{N}{p_{IR}\Delta t}\frac{\sum_{i,j} \sum_{t} {\Delta N}_{S}(t)}{\sum_{i,j} \sum_{t} N_{S}{(t)N}_{I}(t)}$ (5)

where ***N*(*t*)**, ***N_S_*(*t*)** and ***N_I_*(*t*)** represent the total, susceptible and infectious population of agents at time ***t***, respectively. ***ΔN_S_*(*t*)** represents the variation of ***N_S_*** in the integration time step ***Δt*** at time ***t***. A weighed mean was taken to reduce the influence of the fluctuation of data. ***i*** and ***j*** represent the ***i***th trajectory and ***j***th simulation.

**3.3** When the mobility ***v*** is sufficiently large, the basic reproduction number ***R_0_*** can be calculated by mean field theory

$R_{0}=\frac{\pi d^{2}\rho p_{SE}}{p_{IR}}$ (6)

This is close to the ***R_0_*** calculated when v=0.05 m/s.

**3.4** We can define the stable velocity ***v_0_*** such that when ***v*** > ***v_0_***, the epidemic is spreading. We can derive ***v_0_*** from the fitting curve of the basic reproduction number ***R_0_*** we calculated (***R_0_***(***v_0_***) = 1).


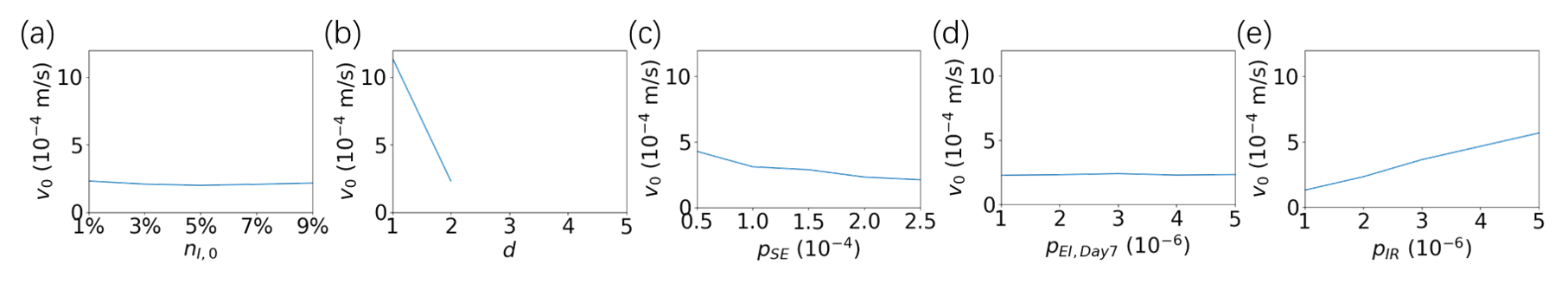


**S3 Fig. The stable velocity *v_0_* influenced by parameters. *v_0_*** is influenced by ***n_I,0_*** (a), ***d*** (b), ***p_SE_*** (c), ***p_EI,Day7_*** (d), and ***p_IR_*** (e). ***v_0_*** is smaller than the smallest velocity of 0.0001 m/s we simulated when ***d*** > 2.

We can also [hypothesis](http://www.baidu.com/link?url=if5slpeCxUIqx_xsbdpW1Y23MD8bWbDRKZkOqjhtwtIryTZ-xhnbXtRMfHWDS2ASG7ehwimk_s32SK-dih_fDWKa5ZKI9-I5VDfAQ8W3OYC) that when $\frac{\pi d^{2}\rho p_{SE}}{p_{IR}}$<1 by mean field theory, the epidemic cannot spread regardless of how large the mobility ***v*** is.

**4 The peak proportion of infections *n_I,max_* and the infection rate *p***

In addition to ***R_0_***, we also calculated the peak proportion of infections in the system, ***n_I,max_***, and the infection rate ***p*** of the system when the epidemic had developed for 40 days to characterize the spread. As shown in **S4** **Fig a and b**, the two descriptors, ***n_I,max_*** and ***p***, exhibit similar parameter dependence with ***R_0_***, i.e., infectivity is positively correlated with ***d***, ***p_SE_***, and ***p_EI,Day7_***, negatively correlated with ***p_IR_***, and less correlated with initial ***n_I,0_***. However, compared with ***n_I,max_*** and ***p***, we found that the basic reproduction number ***R_0_*** is less affected by the randomness of the system and is almost independent of the initial conditions of the system and latent period.


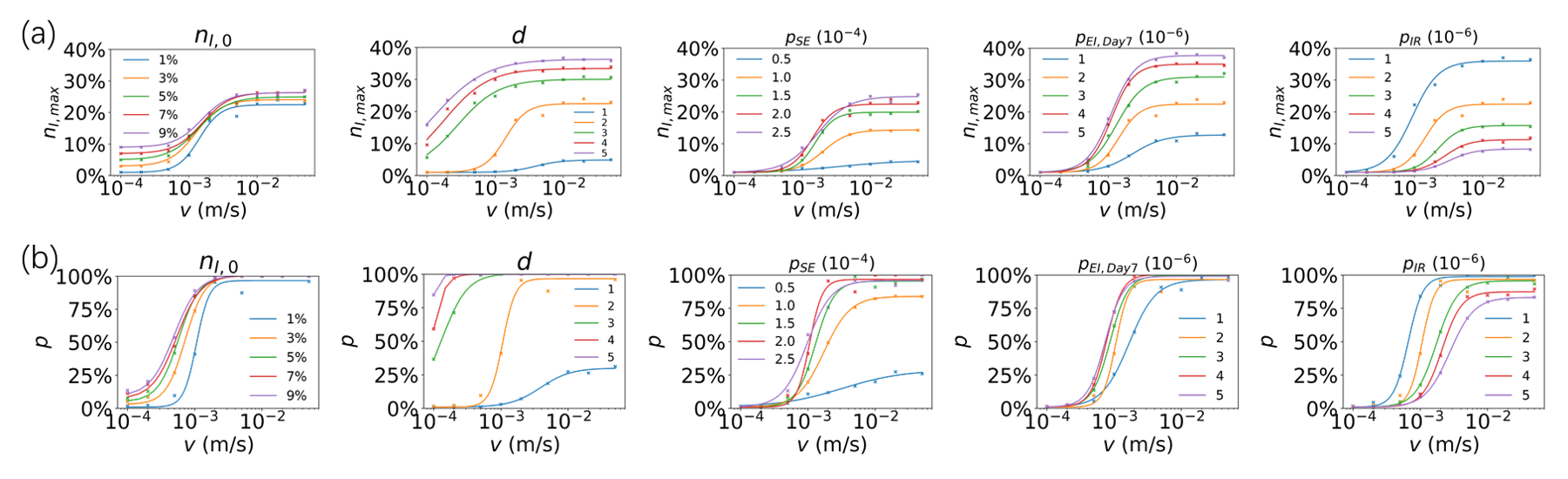


**S4 Fig. Trends in the spread of the epidemic as it had developed for 40 days, influenced by five parameters (*n_I,0_*, *d*, *p_SE_*, *p_EI,Day7_*, and *p_IR_*) and the velocity *v* of agents in the system.** Here, spreading is characterized by the peak proportion of infectious ***n_I,max_*** (a) and the infection rate ***p*** (b) of the system. In each plot, the results corresponding to the five values used for the parameter that changed are represented by five different colored curves. The points represent the calculated data, and the solid line represents the fitting curve to eliminate noise. The horizontal coordinate ***v*** is on a logarithmic scale.

**4.1 The peak proportion of infections *n_I,max_***

We first record the peak number of ***P_I_ N_I,max(i,j)_*** of the ***i***th trajectory, ***j***th simulation in which agents move with a mean velocity ***v,*** and then calculate the peak proportion of infections ***n_I,max_*** by equation 7 over all 25 simulations.

$n_{I,max}=\frac{1}{25}\frac{\sum_{i,j} N_{I,max(i,j)}}{N}$ (7)

**4.2 The infection rate *p***

We first record the infected number of agents when the simulation ends by ***N-N_S_(40day)*** of ***i***th trajectory, ***j***th simulation in which agents move with a mean velocity ***v*** and then calculate the infection rate ***p*** by equation 8 over all 25 simulations.

$p=\frac{1}{25}\frac{\sum_{i,j} (N-N_{S}(40day))}{N}$ (8)

**5** **The critical velocity *v_c_*, slope** ***k*, and saturated reproduction number *R_S_***

**5.1 *R_0_*** shows two stages of variation. In the strong heterogeneity range, ***R_0_*** is proportional to the logarithm of velocity, and in the weak heterogeneity range, ***R_0_*** reaches saturation. Therefore, we fit the basic reproduction number ***R_0_*** by the function

$f(a,b,c,d)=a\ln(\frac{v}{b})e^{-dv}+c(1-e^{-dv})$ (9)

where ***a***, ***b***, ***c***, and ***d*** are undetermined coefficients. This function performs close to $a\ln(\frac{v}{b})$ when ***v*** is close to 0, while it reaches saturation when ***v*** tends to ∞.

**5.2** We can obtain the function ***R_0_*(*v*)** by fitting the data of ***R_0_*** using equation 9. Then, we define the saturated reproduction number ***R_S_***

$R_{S}=\lim_{v\to\infty} R_{0}(v)$ (10)

The critical velocity ***v_c_*** of ***R_0_*** is defined as

$R_{0}(v_{c,R_{0}})=0.9R_{S}$ (11)

The slope ***k*** is defined as

$k=\max_{v\in[v_{min},v_{max}]} \frac{dR_{0}(v)}{dv}$ (12)

where ***v_min_***=10^-4^ m/s and ***v_max_***=0.05 m/s.

**6 Heterogeneity variable ζ**

**6.1** We record the total number of all individuals within radius ***d*** of all ***P_I_*** at time ***t N´(t)***, the total number of ***P_S_*** within radius ***d*** of all ***P_I_*** at time ***t N_S_´(t)***, and the number of ***P_S_*** at time ***t N_S_(t)*** of ***i***th trajectory, ***j***th simulation in which agents move with a mean velocity ***v***. Then, we calculate the relative neighbor concentration ***RNC_S_*** and obtain heterogeneity variable ***ζ*** by equation 13 over all 25 simulations.

${\zeta=1-RNC}_{S}=1-<\frac{N_{S}^{'}/N^{'}}{N_{S}/N}>=1-\frac{\sum_{i,j} \sum_{t} N_{S}^{'}(t)*N}{\sum_{i,j} \sum_{t} N'(t)*N_{S}(t)}$ (13)

**6.2** The relative neighbor concentration ***RNC_S_*** performs similarly to the basic reproduction number ***R_0_***. We can obtain the function ***RNC_S_* (*v*)** by fitting the ***RNC_S_*** data using equation 9, in which we set ***c***=1. The critical velocity ***v_c_*** of ***ζ*** is defined as

${RNC}_{S}(v_{c,\zeta})=0.9(\zeta=0.1)$ (14)

**7 The required contact time** ***t_SE_*, the number of contacts before infection** ***T_SI_*, and the mean contact time before infection** ***ω_SI_***

We sift the contact data in which the central agent is ***P_S_*** and its neighbor is ***P_I_*** of ***i***th trajectory, ***j***th simulation in which agents move with a mean velocity ***v***. The sifted data are classified by the central particle. ***t_SE_***, ***T_SI_*** and ***ω_SI,_*** whose central particle is agent ***n,*** are calculated by equations 15, 16, and 17, respectively. A statistical analysis will be carried out on ***t_SE_***, ***T_SI_*** and ***ω_SI_*** over all 25 simulations.

$t_{SE(n)}=\sum_{Index(CP)=n,CP\in P_{S},NP\in P_{I}} \omega$ (15)

$T_{SI(n)}=\sum_{Index(CP)=n,CP\in P_{S},NP\in P_{I}} 1$ (16)

$\omega_{SI(n)}=\frac{t_{SE(n)}}{T_{SI(n)}}=\frac{\sum_{Index(CP)=n,CP\in P_{S},NP\in P_{I}} \omega}{\sum_{Index(CP)=n,CP\in P_{S},NP\in P_{I}} 1}$ (17)

where ***CP*** represents the central particle, ***NP*** represents the neighboring particle and ***Index (CP)*** represents the index of the central particle.

A susceptible agent ***P_S_***, which is considered a central particle, with index ***n*** may need multiple contacts with ***P_I_*** to get infected; therefore, ***t_SE_*** represents the sum of the duration of these contacts, and ***t_SE_(n)*** is the ***t_SE_*** for ***P_S_*** with index ***n***. ***T_SI_*** represents the total number of these contacts. ***ω_SI_*** represents the average duration of contact. We calculate ***t_SE_***, ***T_SI_*** and ***ω_SI_*** for each central particle.
