# Supplementary material for: Spatial heterogeneity and infection patterns on epidemic transmission disclosed by a combined contact-dependent dynamics and compartmental model
Source: PLoS One. 2023 Jun 13;18(6):e0286558. doi: 10.1371/journal.pone.0286558 (PMC10263307; doi:10.1371/journal.pone.0286558)
Supplement: Attachment — Submitted filename: Comments-PLOSONE.pdf [file pone.0286558.s002.pdf]

Title: Spatial heterogeneity and infection patterns on epidemic transmission disclosed by a combined contact-dependent dynamics and compartmental model.

Comments: In this paper, the authors proposed a modified SEIR-CDD model, where SEIR model to describe infection between individuals and CDD model designed to characterize the stochastic motion of individuals within a community. The exposure time ω and mean free time τ are discussed. Meanwhile, the epidemic transmission characteristics described by the SEIR-CDD model, the calculated reproduction number R and spatial heterogeneity ζ are obtained. And the effects of motion parameters (such as speed) on these characteristics are obtained. The bipartite variation of R with the strength of spatial heterogeneity is discussed. The research content of the paper is interesting and somewhat innovative.

The details of my comments are as follows:

(1) In page 4, line 7, "consists" should be "consisted", such problems should be paid attention to.

(2) In page 5, line 9, "SEIR stands for susceptible, exposed, infectious, and recovered," should be "S, E, I and R stand for susceptibility, exposure, infection and recovery, respectively,"; In page 5, line -12 and -11, what is the mean "be ∼7 days"?

(3) In page 7, the following line (i.e., where) of equation 2 should not be indented; In page 7, line 8, and the last line of the note in Figure 2, "a mean of 0" should be "a mean of 0°".

(4) How to choose p_{EI} like $p_{EI,Day7} * \frac{2}{1+e^{3(T-7)}}$ for COVID-19, please explain.

(5) For the modified SEIR model, it ignores some factors, such as death due to disease, repeated infection, vaccination, asymptomatic infected persons, etc., can it describe the transmission law and transmission mechanism of epidemic diseases, especially COVID-19? It can be seen that the author's model must be based on a basic assumption in order to be valid. Population dynamics such as birth, natural death and movement are not considered, which means that a closed environment is considered and diseases are assumed to change significantly over time than birth and death over time, so that the latter can be ignored. Obviously, from the model and simulation point of view, this is easy to do but does it miss some important information? Should the authors elaborate further on how to consider in this section?

(6) In the CDD-SEIR model, the motion of the agents consists of alternating individual motions and contact between individuals (thus changing the motion) in an ensemble of individuals. But as shown in Figure 3a, please

notice that the region has a boundary, how does it move on the boundary? Does it change direction when it hits the boundary? How can it change?

(7) In page 9, line 12, “$p_{SE} = 2 * 10^{-4,}$” should be “$p_{SE} = 2 * 10^{-4}$,”

(8) In page 13, 10, 6 and 5, the format of “(equation 4)”, “(Equation 3)”, “(equation 2)” and “(equation 1)” should be the same format.

(9) In page 12, in the first line of the note in Figure 5, “different p_{SEs}” should be “different p_{SE}”.

(10) For R, there are many different expressions in the passage, such as: “the reproduction number R” (In page 11), “the propagation number R” (In page 15, line 2), and “the transmission number R” (In page 17, line 4). The author should give the same expression. Similar to the basic reproduction number R_0, R in this paper is an important value, and its meaning should be further elaborated and verified by numerical simulation if possible.

(11) As shown in Figure 6a, the reproduction number R influenced by parameter is studied using the control variable method. Can local sensitivity analysis or global sensitivity analysis be performed similar to the typical SEIR model? The authors are recommended to add sensitivity analysis for R if it is possible for them.

(12) For Figure 6c, that does not seem to obtain that “Specifically, v_c is negatively correlated with d, positively correlated with p_{SE}” (In page 13, line 5). The image of it shows that it is not monotonous, and is related to the value range of parameters. If the value range of parameters is expanded, will more complex thing appear? The purpose of using logarithmic coordinates in drawing is to reduce the data dispersion or density caused by excessive scale. Here, the correlation between parameters is mainly looked at. The authors can try linear coordinates to see which coordinates will be clearer.

(13) The authors obtained some interesting conclusions through numerical simulation. However, from the perspective of the reliability of the research, in the numerical simulation stage, using the existing parameter values in other literatures and the parameter values assumed by oneself may lead to the experimental deviation due to the inaccurate parameter values and lack of authenticity. The authors can use the model to fit the real data of COVID-19, which can not only ensure the accuracy of parameter values and verify the effectiveness of the model, but also obtain some further conclusions and enlightenment.

(14) Please unify the reference format and check the reference 15 and 32 again.

(15) For Supporting Information, in page 6, please explain the signs in equations 8, 9 and 10; In the fourth line from the bottom of the note in Figure S1, “(b)” should be “(c)”; In page 3, “200’000 times” and “200000

times" should be unified; For some key parameters, the authors should give the simple derivations or explanations.
