# Supplementary material for: Spatial heterogeneity and infection patterns on epidemic transmission disclosed by a combined contact-dependent dynamics and compartmental model
Source: PLoS One. 2023 Jun 13;18(6):e0286558. doi: 10.1371/journal.pone.0286558 (PMC10263307; doi:10.1371/journal.pone.0286558)
Supplement: Attachment — Submitted filename: Response_230406.docx [file pone.0286558.s003.docx]

**Responses and changes to Reviewers' comments and suggestions**

We appreciate the valuable comments and suggestions by the two reviewers to make our manuscript more rigorous. We checked the manuscript carefully, made changes as suggested, and corrected the typos. Our responses and content changes item by item are listed below. The comments and suggestions are in italic style, and our responses are colored in blue.

***Reviewer 1:***

***Title:*** *Spatial heterogeneity and infection patterns on epidemic transmission disclosed by a combined contact-dependent dynamics and compartmental model.*

***Comments****: In this paper, the authors proposed a modified SEIR-CDD model, where SEIR model to describe infection between individuals and CDD model designed to characterize the stochastic motion of individuals within a community. The exposure time* ***ω*** *and mean free time* ***τ*** *are discussed. Meanwhile, the epidemic transmission characteristics described by the SEIR-CDD model, the calculated reproduction number R and spatial heterogeneity* ***ζ*** *are obtained. And the effects of motion parameters (such as speed) on these characteristics are obtained. The bipartite variation of* ***R*** *with the strength of spatial heterogeneity is discussed. The research content of the paper is interesting and somewhat innovative.*

*The details of my comments are as follows:
(1) In page 4, line 7, "consists" should be "consisted", such problems should be paid attention to.*

Response: Thank you. We have carefully corrected these typos.

*(2) In page 5, line 9, "SEIR stands for susceptible, exposed, infectious, and recovered," should be "S, E, I and R stand for susceptibility, exposure, infection and recovery, respectively,"; In page 5, line -12 and -11, what is the mean "be ∼7 days"?*

Response: Thank you. We have revised it as suggested by the reviewer. We have also changed "be ∼7 days" to "be approximately 7 days" (on page 7).

*(3) In page 7, the following line (i.e., where) of equation 2 should not be indented; In page 7, line 8, and the last line of the note in Figure 2, "a mean of 0" should be "a mean of 0◦".*

Response: Thank you. We have revised it (on page 10) as suggested by the reviewer.

*(4) How to choose* ***p_EI_*** *like* $p_{EI}=p_{EI,Day7}*\frac{2}{1+e^{3(T-7)}}$ *for COVID-19, please explain.*

Response: We used the parameter ***p_EI_*** to characterize the probability distribution of an agent changing from exposed (***E***) to infected (***I***) during the latent period. The latent period is the time from exposure to becoming infectious. In the literature, log-normal, Weibull, gamma distributions as well as empirical distributions based on statistics of contact-tracing data have been used to characterize the latent period (please see, for example, *N. Engl. J. Med.*, **2020**, *18*, 1708-1720; *N. Engl. J. Med.*, **2020**, *13*, 1199-1207; *Biometrics*, **2021**, *77*, 929-941; *Nonlinear Dyn.*, **2021**, *106*, 1347-1358). One possible reason for using different strategies is that latent period data are difficult to obtain, and therefore it is difficult to determine the appropriate functional form.

The latent period for COVID-19 was estimated to be approximately 7 days and concentrated in about 4-7 days in the literature. In this work the parameter ***p_EI_*** was defined as:

$${p_{EI}=\{}_{p_{EI,Day7}*\frac{2}{1+e^{3\left( T-7 \right)}}, 4\leq T<10}^{0, T<4}$$

where ***T*** is in the unit day, and ***p_EI,Day7_*** is the conversion probability of the 7^th^ day of the latent period with its value shown in **Table 1**. According to the above representation, within 0-4 days, ***P_E_*** only acts as the virus carrier and does not have the ability to infect others; within 4-10 days, ***P_E_*** can transform with the probability ***p_EI_*** to ***P_I_*** that can transmit the virus; otherwise, it remains as ***P_E_***. After 10 days, ***P_E_*** that still does not convert to ***P_I_*** is classified as ***P_R_***. Meanwhile, ***P_I_*** can be transformed into ***P_R_*** with the probability ***p_IR_***.

We have revised the equation 1 and relevant paragraphs on pages 7-8.

*(5) For the modified SEIR model, it ignores some factors, such as death due to disease, repeated infection, vaccination, asymptomatic infected persons, etc., can it describe the transmission law and transmission mechanism of epidemic diseases, especially COVID-19? It can be seen that the author’s model must be based on a basic assumption in order to be valid. Population dynamics such as birth, natural death and movement are not considered, which means that a closed environment is considered and diseases are assumed to change significantly over time than birth and death over time, so that the latter can be ignored. Obviously, from the model and simulation point of view, this is easy to do but does it miss some important information? Should the authors elaborate further on how to consider in this section?*

Response: We appreciate the reviewer for raising a very important question, and we fully agree with the reviewer that factors such as vaccination may play an important role in the spread of epidemics. In the current work, we considered only four types of agents (S, E, I, and R) without further inclusion of additional factors based on the following considerations:

(1) In the current work, we proposed a random motion model that can characterize human activities and, as a result, obtained the basic patterns of evolution of the four basic types of agents in epidemic transmission. Introducing additional factors would increase the complexity of the system and thus may obscure the most important information. After understanding the dynamics of the four types of individuals, we can further explore the influence of other factors on the spread of the epidemic by means of control variables.

(2) Other factors such as repeated infections, and vaccination, can be easily added to the current model (by adding types of agents). And factors such as death can also be implemented in the current model by removing particles from the system or eliminating their movement. This is also our proposed research work at a later stage.

(3) As pointed out by the reviewer, our current model describes a closed system and short time scales (e.g., days to tens of days). Therefore, it may be less applicable to describe changes in epidemic transmission due to birth.

The relevant information has been added on page 11-13 of the revised MS.

*(6) In the CDD-SEIR model, the motion of the agents consists of alternating individual motions and contact between individuals (thus changing the motion) in an ensemble of individuals. But as shown in Figure 3a, please notice that the region has a boundary, how does it move on the boundary? Does it change direction when it hits the boundary? How can it change?*

Response: We apologize for not making this more precise. We used periodic boundary conditions in the simulations. Therefore, when an agent passes through one side of the unit cell, it re-appears on the opposite side with the same velocity.

*(7) In page 9, line 12, "****p_SE_*** *= 2 ∗ 10^-4,^" should be "****p_SE_*** *= 2 ∗ 10^-4^,"*

Response: Thank you. We have corrected it (page 14).

*(8) In page 13, 10, 6 and 5, the format of "(equation 4)", "(Equation 3)", "(equation 2)" and "(equation 1)" should be the same format.*

Response: Thank you. We have revised it.

*(9) In page 12, in the first line of the note in Figure 5, "different* ***p_SEs_****" should be "different* ***p_SE_****".*

Response: Thanks. We have revised it.

*(10) For R, there are many different expressions in the passage, such as: "the reproduction number* ***R****" (In page 11), "the propagation number* ***R****" (In "page 15, line 2), and "the transmission number* ***R****" (In page 17, line 4). The author should give the same expression. Similar to the basic reproduction number* ***R_0_****,* ***R*** *in this paper is an important value, and its meaning should be further elaborated and verified by numerical simulation if possible.*

Response: Our originally used ***R*** differs from the commonly used ***R_0_*** (basic reproduction number) in definition, which may cause misunderstanding. To be consistent with the definition used in the literature, we have changed to use ***R_0_*** in the revised manuscript.

Similar to the SEIR model,

$$\frac{dN_{S}}{dt}=\beta\frac{N_{I}}{N}N_{S}$$

$$\frac{dN_{R}}{dt}=\gamma N_{I}$$

where ***N_S_***, ***N_I_***, ***N_R_*** and ***N*** represent the susceptible, infectious, recovered and total populations of agents, respectively, and ***β*** and ***γ*** represent the transmission rate and recovery rate, respectively, and therefore, ***R_0_*** can be calculated by

$$R_{0}=\frac{\beta}{\gamma}$$

In the revised MS, we calculated ***R_0_*** similarly by referring to the idea of the mean field method,

$$R_{0}=\frac{\beta}{p_{IR}}=\frac{N}{p_{IR}dt}\frac{{dN}_{S}}{N_{S}N_{I}}=\frac{N}{p_{IR}\Delta t}<\frac{{\Delta N}_{S}}{N_{S}N_{I}}>$$

where ***ΔN_S_*** represents the variation in ***N_S_*** in the integration time step Δ***t***. The obtained ***R_0_*** in this work is consistent with data estimated from mean field theory.

The relevant information has been added to pages 17-18 in the revised MS.

*(11) As shown in Figure 6a, the reproduction number* ***R*** *influenced by parameter is studied using the control variable method. Can local sensitivity analysis or global sensitivity analysis be performed similar to the typical SEIR model? The authors are recommended to add sensitivity analysis for* ***R*** *if it is possible for them.*

Response: In the revised MS, we have replaced the reproduction number ***R*** with the basic reproduction number ***R_0_***. The influences of parameters, such as ***n_I,0_***, ***d***, ***p_SE_***, ***p_EI,Day7_***, and ***p_IR_***, on ***R_0_*** are shown in **Fig 6a**. To be specific, we changed one variable and kept others at their baseline values (***n_I,0_*** = 1%, ***d*** = 2, ***p_SE_*** = 2*10^-4^, ***p_EI,Day7_*** = 2*10^-6^, ***p_IR_*** = 2*10^-6^) and studied the influence of parameters. This control variable method is consistent with the idea of local sensitivity analysis, for example, those reported in the literature (*IEEE J Biomed Health Inform*, **2022**, *26*, 2458-2468).

However, we failed to carry out the global sensitivity analysis, mainly because more extensive sampling of the parameters is needed for global sensitivity analysis. In the current model, we set the integration step size of the equation of motion to 1 second to ensure the integration accuracy without missing the events during the individual motion. Thus, it takes ~1 month or more to accumulate a 40-day trajectory of a system containing hundreds of agents. To obtain a reliable result for the global sensitivity analysis, we need a huge number of such data points (for example, for n parameters, each test m points, we should have a total number of m^n^ points), but we only obtained approximately several dozen points in our previous work, mainly due to the limitation from the computational cost. Therefore, we could not present the results for the global sensitivity analysis. We sincerely hope that the reviewers will take this into consideration and agree with us.

*(12) For Figure 6c, that does not seem to obtain that "Specifically,* ***v_c_*** *is negatively correlated with* ***d****, positively correlated with* ***p_SE_****" (In page 13, line 5). The image of it shows that it is not monotonous, and is related to the value range of parameters. If the value range of parameters is expanded, will more complex thing appear? The purpose of using logarithmic coordinates in drawing is to reduce the data dispersion or density caused by excessive scale. Here, the correlation between parameters is mainly looked at. The authors can try linear coordinates to see which coordinates will be clearer.*

Response: Based on the reviewer’s suggestion, we performed additional simulations (for ***d*** = 1.5 and 2.5). Additionally, we replaced ***R*** with ***R_0_*** to replot **Fig. 6C** with linear coordinates (instead of the originally used logarithmic coordinates).

**Fig R1**. The critical velocity ***v_c_*** of ***R_0_*** is influenced by ***d***.

We noticed that ***v_c_*** is almost independent of ***d*** compared with the deviation of ***n_I,0_***, which is different from what we observed before (**Fig. 6C**). However, ***v_c_*** is positively correlated with ***p_SE_.*** Meanwhile, we also noticed that the basic reproduction number ***R_0_*** and the heterogeneity variable ***ζ*** show good agreement (**Fig. 6C**).

The relevant information can be found on pages 19-21 of the revised MS.

*(13) The authors obtained some interesting conclusions through numerical simulation. However, from the perspective of the reliability of the research, in the numerical simulation stage, using the existing parameter values in other literatures and the parameter values assumed by oneself may lead to the experimental deviation due to the inaccurate parameter values and lack of authenticity. The authors can use the model to fit the real data of COVID-19, which can not only ensure the accuracy of parameter values and verify the effectiveness of the model, but also obtain some further conclusions and enlightenment.*

Response: Thank you for your advice. We have considered using real data as the reference and fitting the results with simulations to explore the reliability of our CDD-SEIR model.

However, limited by the current model setup, such a direct comparison is not easy: first, we used 1 s as the integration step of the equation of motion to not miss any of the exposure events, and thus currently, the model can only handle a few hundred agents in a few tens of days within an affordable computational cost; second, for a system containing hundreds of particles, a single initial infected agent already corresponds to a very high initial infection rate (e.g., 1 in 200 agents corresponds to an initial infection rate of 2.5%), while the ratio in the real situation is often less than 1 in 10,000. Therefore, the present work lies mainly in providing a simple model with clear physical meaning to study the dynamics of epidemic transmission.

The relevant information has been added to pages 12-13 of the revised MS.

*(14) Please unify the reference format and check the reference 15 and 32 again.*

Response: Thank you. We have unified the reference format and removed duplicates.

*(15) For Supporting Information, in page 6, please explain the signs in equations 8, 9 and 10;*

Response: We apologize for not making this more precise. We have revised equations 8-10 (now equations 15-17) where ***CP*** represents the central particle, ***NP*** represents the neighboring particle and ***Index (CP)*** represents the index of the central particle.

A susceptible agent ***P_S_***, which is considered as a central particle, with index ***n*** may need multiple contacts with ***P_I_*** to get infected; therefore, ***t_SE_*** represents the sum of the duration of these contacts, and ***t_SE(n)_*** is the ***t_SE_*** for ***P_S_*** with the index ***n***. ***T_SI_*** represents the total number of contacts. ***ω_SI_*** represents the average duration of contacts.

We calculate ***t_SE_***, ***T_SI_*** and ***ω_SI_*** for each central particle using the following equations:

$t_{SE(n)}=\sum_{Index(CP)=n,CP\in P_{S},NP\in P_{I}} \omega$	(15)

$T_{SI(n)}=\sum_{Index(CP)=n,CP\in P_{S},NP\in P_{I}} 1$	(16)

$\omega_{SI(n)}=\frac{t_{SE(n)}}{T_{SI(n)}}=\frac{\sum_{Index(CP)=n,CP\in P_{S},NP\in P_{I}} \omega}{\sum_{Index(CP)=n,CP\in P_{S},NP\in P_{I}} 1}$	(17)

The relevant information has been added to page 10 of the SI.

*(16) In the fourth line from the bottom of the note in Figure S1, "(b)" should be "(c)"; In page 3, "200’000 times" and "200000 times" should be unified;*

Response: We have corrected these typos.

*(17) For some key parameters, the authors should give the simple derivations or explanations.*

Response: We thank the reviewers for pointing this out. We have read the article carefully and found that the description for equation 1 in SI was unclear.

$$\omega=\frac{2r_{c}cos<\vec{v^{'}},\vec{e_{x}}>}{v^{'}}=\frac{2r_{c}v_{x}^{'}}{{\vec{v^{'}}}^{2}}$$

where $\vec{\boldsymbol{e}_{\boldsymbol{x}}}$ represents the unit vector of the ***X***-axis and $\boldsymbol{<}\vec{\boldsymbol{v}^{\boldsymbol{'}}}\boldsymbol{,}\vec{\boldsymbol{e}_{\boldsymbol{x}}}\boldsymbol{>}$ represents the angle between vectors $\vec{\boldsymbol{v}^{\boldsymbol{'}}}$ and $\vec{\boldsymbol{e}_{\boldsymbol{x}}}$.

The calculation of the basic reproduction number ***R_0_***, critical velocity ***v_c_***, slope ***k*** and saturated reproduction number ***R_S_*** have been updated in the revised SI file.

***Reviewer 2:***

*(1) The paper investigates a SEIR compartmental model of epidemic dynamics. Basically it considers a population of random walkers ('agents') in a box where a transmission of the disease takes place in case of contact of an infected and a susceptible agent. The simulation is performed purely numerically, however, not sufficient details are provided to repeat these simulations. Some more information on the numerics should be included.*

Response: We thank the reviewers for pointing out this problem. Here are more details about the simulations:

We set the number of agents ***N***, the box size ***L***, the contact distance ***r_c_*** and the average velocity ***v*** of agents. Then we ran simulations with the CDD model to generate trajectories of each agent. To enhance sampling and reduce the randomness caused by the numerical simulations, we accumulated five parallel trajectories for each ***v***.

Then, the rest of the propagation parameters including the initial proportion of ***P_I_*** ***n_I,0_***, the airborne transmission distance ***d***, the infection probability ***p_SE_***, the conversion probability of Day 7 from the exposed agent (***P_E_***) to the infectious agent (***P_I_***) ***p_EI,Day7_***, and the recovery probability ***p_IR_*** were chosen by the control variable method (**Table 1**). We postprocessed each trajectory using the modified SEIR model to obtain 5 independent modes of transmission. Therefore, for each set of parameters, the final reported data are the average of 5*5=25 sets of simulation results.

The relevant information has been added to pages 13-14 of the revised MS.

*(2) It seems to me that the 'reproduction number* ***R****' the authors work with corresponds to the 'effective reproduction number* ***Re****' (number of new infections generated by one infectious agent, whereas* ***R_0_*** *indicates this number when one infectious walker is present in a purely susceptible population). The authors should clarify this issue ad give a formal explicit definition of their '****R*** *and better explain its precise meaning'.*

Response: Our originally used ***R*** differs from the commonly used ***R_0_*** (basic reproduction number) in definition, which may cause misunderstanding. To be consistent with the definition used in the literature, we have changed to use ***R_0_*** in the revised manuscript.

Similar to the SEIR model,

$$\frac{dN_{S}}{dt}=\beta\frac{N_{I}}{N}N_{S}$$

$$\frac{dN_{R}}{dt}=\gamma N_{I}$$

where ***N_S_***, ***N_I_***, ***N_R_*** and ***N*** represent the susceptible, infectious, recovered and total populations of agents, respectively, and ***β*** and ***γ*** represent the transmission rate and recovery rate, respectively, and therefore, ***R_0_*** can be calculated by

$$R_{0}=\frac{\beta}{\gamma}$$

In the revised MS, we calculated ***R_0_*** similarly by referring to the idea of the mean field method,

$$R_{0}=\frac{\beta}{p_{IR}}=\frac{N}{p_{IR}dt}\frac{{dN}_{S}}{N_{S}N_{I}}=\frac{N}{p_{IR}\Delta t}<\frac{{\Delta N}_{S}}{N_{S}N_{I}}>$$

where ***ΔN_S_*** represents the variation in ***N_S_*** in the integration time step Δ***t***. The obtained ***R_0_*** in this work is consistent with data estimated from mean field theory.

The relevant information has been added to pages 18-19 in the revised MS.

*(3) The paper is actually well written and the results are rather interesting. It would be nice to see a short stability analysis under which condition an epidemics is spreading (such as* ***R_0_****>1 in standard models). What are the pertinent parameters that makes the heathy initial state (one infectious agent in a susceptible population) unstable (such as* ***R_0_*** *>1)?*

Response: We appreciate the comments from the reviewer.

Regarding the stability analysis, if we understand correctly, the requirement for a steady-state solution for an epidemic model, such as the SIR model, to emerge is that there is a continuous addition of susceptible agents (e.g., after considering the effect of birth rate). Please see, for example, *J. Phys.: Conf. Ser.* **2022**, *2199*, 012035; *Math. Probl. Eng.* **2022**, *2022*, 6962160. In the current work, we did not consider such effects, so the number of susceptible agents (***P_S_***) decreases continuously with time and the number of recovered agents (***P_R_***) increases monotonically. Seemingly, there is no endemic equilibrium in our model. Additionally, we cannot derive precise differential equations for populations because of the complexity of motion. Therefore, the present model is temporarily unavailable for stability analysis.

**Fig R2**. The range of five parameters (***n_I,0_***, ***d***, ***p_SE_***, ***p_EI,Day7_***, and ***p_IR_***) for ***R_0_*** < 1 (black dot) and ***R_0_*** ≥ 1 (red dot) calculated at different velocities based on the CDD-SEIR model.

Regarding the pertinent parameters in our model, as mentioned in our response to the first question, we used a control-variable approach to sampling, i.e., varying one of the parameters ***n_I,0_***, ***d***, ***p_SE_***, ***p_EI,Day7_***, ***p_IR_***, while keeping the other parameters unchanged. We selected five values for each changed parameter and performed calculations at nine different velocities ***v***. Our discussion is mainly based on the results obtained for the control variables.

We calculated ***R_0_*** for the above parameter cases, as shown in **Fig 6a** in the MS. We counted these parameters to obtain the parameter values ***R_0_*** < 1 (black dots in **Fig R2**) as well as ***R_0_*** > 1 (red dots in **Fig R2**). Clearly, as the velocity ***v*** increases, an increase in ***d***, ***p_SE_*** or a decrease in ***p_IR_*** will be more likely to cause a susceptible agent (***P_S_***) to become infected (***R_0_*** changes from less than 1 to greater than 1), while ***n_I,0_*** and ***p_EI,Day7_*** have little effect on ***R_0_***.

To further explore the parameters under which ***R_0_*** > 1 is realized, we can define ***v_0_*** that satisfies ***R_0_***(***v_0_***) = 1. Then, with the corresponding parameters, it is clear that ***R_0_*** > 1 when ***v*** > ***v_0_*** (**Fig S3 in SI**).

*(4) In a revision the authors should also discuss possible connections to existing SEIR and other compartment models, such as a random walker's approach PhysRevE.105.024205 and existing SEIR models* [*https://doi.org/10.1038/s41592-020-0856-2*](https://doi.org/10.1038/s41592-020-0856-2) *)*

Response: We appreciate the reviewer for bringing us these insightful papers. The two papers were cited as Ref. 37 and 38. We have made some comparisons as follows, which can be found on pages 25-27 of the revised MS:

“Various epidemiological models that consider multiple types of compartments beyond the basic SIR framework have been proposed. In this study, we compare our CDD-SEIR method to two recently reported methods. One is a random walker’s approach that incorporates the memory effect into a compartment model [Ref. 37], where recovered agents are assumed to stay immune for a random duration. In contrast, our work considers the scenario where the susceptible agents become carriers after being infected and combines a modified SEIR model with contact-dependent dynamics. Both methods are based on the traditional SIR model and explore the influence of other factors on space-time dynamics of epidemics. The random walker’s method is efficient for characterizing epidemics in the long term, while our CDD-SEIR model introduces more dynamical details by considering patterns of contacts. As the two methods consider different impacts of other factors on disease transmission, direct comparison of results is challenging. Future studies can further incorporate an immunity impact on transmission in our model.

The SEIRS model considers the effects of birth, death, and incubation period on infectious disease dynamics [Ref. 38]. Both this model and our work take into account the latency effect, although in different ways. However, the SEIRS model integrates multiple influences, such as the length of the latent period, the various routes of transmission, and age-specific transmission. Additionally, the introduction of repeated infection in the SEIRS and random walker's models enables the epidemic equilibrium, allowing for longer timescale characterization of the disease spreading. While our CDD-SEIR model can incorporate these factors by adding agent types to simulate more realistic scenarios, we primarily focused on the impact of contact due to motion on epidemic transmission. Although some details of the epidemic transmission process are excluded, our CDD-SEIR model captures the fundamental information and physical laws.”

--------------------------------

This is the end of the file. We would like to sincerely thank the two reviewers for their comments and suggestions, which helped us to improve this work.

--------------------------------
